# Mechanism of Azalomycin F_5a_ against Methicillin-Resistant* Staphylococcus aureus*

**DOI:** 10.1155/2018/6942452

**Published:** 2018-01-21

**Authors:** Li Xu, Xuejie Xu, Ganjun Yuan, Yimin Wang, Yunqiu Qu, Erxiao Liu

**Affiliations:** ^1^College of Bioscience and Bioengineering, Jiangxi Agricultural University, Nanchang, Jiangxi 330045, China; ^2^Affiliated Hospital of Jiangxi Agricultural University, Nanchang, Jiangxi 330045, China

## Abstract

To investigate the mechanism of azalomycin F_5a_ against methicillin-resistant* Staphylococcus aureus *(MRSA), the conductivity of MRSA suspension and the adenylate kinase activity of MRSA culture were determined with the intervention of azalomycin F_5a_, which were significantly increased compared to those of blank controls. This inferred that azalomycin F_5a_ could lead to the leakage of cellular substances possibly by increasing permeability to kill MRSA. As phospholipid bilayer was mainly responsible for cell-membrane permeability, the interaction between azalomycin F_5a_ and cell-membrane lipids was further researched by determining the anti-MRSA activities of azalomycin F_5a_ combined with cell-membrane lipids extracted from test MRSA or with 1,2-dipalmitoyl-*sn*-glycero-3-phospho-glycerol (DPPG) for possible molecular targets lying in MRSA cell-membrane. The results indicated that the anti-MRSA activity of azalomycin F_5a_ remarkably decreased when it combined with membrane lipids or DPPG. This indicated that cell-membrane lipids especially DPPG might be important targets of azalomycin F_5a_ against MRSA.

## 1. Introduction

Azalomycin F, a complex of polyhydroxy macrolides including three main components azalomycins F_5a_, F_4a_, and F_3a_, was first isolated from the broth of* Streptomyces hygroscopicus *var*. azalomyceticus* [1, 2]. Unlike macrolide antibiotics as erythromycin and polyene macrolides as amphotericin B, it presented significant antimicrobial and antifungal activities [1–3]. As Sugawara reported, azalomycin F could lead to the leakage of cellular substances to kill* Bacillus subtilis*, while detailed mechanism had not been further reported because their chemical structures were not clear at that time [4]. Following studies indicated that many streptomycetes such as* S. hygroscopicus* and* S. malaysiensis* also had great potential to produce these compounds [3, 5]. Recently, twelve 36-membered macrocyclic lactones including azalomycins F_5a_, F_4a_, and F_3a_ were also isolated from the broth of* Streptomyces *sp. 211726, and their planar structures and relative configurations were elucidated by us in 2011 to 2013 [6–8]. Considering the research and development of new antimicrobial drugs were desperate [9], the inhibitory activities of azalomycins F_5a_, F_4a_, and F_3a_ against multidrug-resistant organisms were further tested, and the results showed they had significant antagonistic activities to methicillin-resistant* Staphylococcus aureus* (MRSA) and vancomycin-resistant* Enterococci* [10]. Moreover, 23-demalonyl azalomycin F obtained by hydrolysis of azalomycin F presented more remarkable anti-MRSA activity and better aqueous solubility and stability than azalomycin F [11]. Thereby, azalomycin F_5a_ as a representative of 36-membered macrolides was worthy of further researching, especially for its anti-MRSA mechanism.

## 2. Materials and Methods

### 2.1. MRSA Strain

MRSA ATCC 33592 obtained from American Type Culture Collection, Manassas, VA, USA, was inoculated in 20 mL of Mueller Hinton Broth (MHB) (Haibo Biotechnology Co., Ltd., Qingdao, China) and cultured at 37°C for 24 h on a rotary shaker (160 rpm). The cultures were diluted with MHB (1 : 100) and were incubated at 37°C for 8 h on a rotary shaker (160 rpm) to obtain MRSA cultures at exponential phase (approx. 1 × 10^8^ cfu/mL) for following experiments.

### 2.2. Azalomycin F_5a_

**Figure 1:**
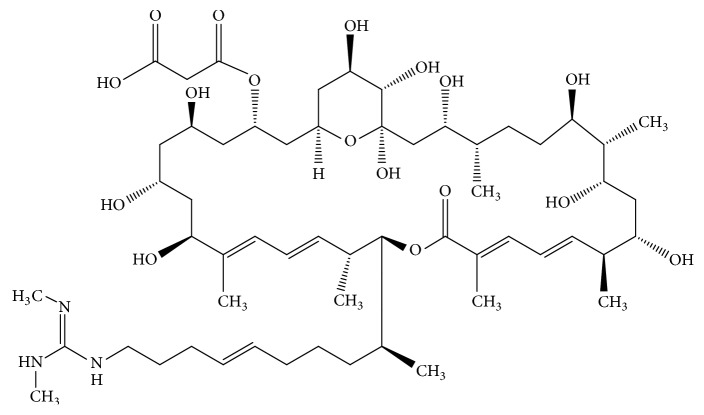
Chemical structure of azalomycin F_5a_.

Azalomycin F_5a_ (Figure 1) was isolated from the broth of* Streptomyces hygroscopicus *var*. azalomyceticus* according to our previous method [6], and its purity (98.2%) was analyzed by Waters 2695 Alliance HPLC System. It was dissolved in DMSO to obtain the concentration of 2048 *μ*g/mL before use and made sure that the concentration of DMSO was less than 1.6% in test system. The minimum inhibitory concentration (MIC) of azalomycin F_5a_ against MRSA ATCC 33592 was 4.0 *μ*g/mL, which was determined in our published work [12].

### 2.3. Conductivity of MRSA Suspension

One hundred milliliters of MRSA cultures at exponential phase was centrifuged for 10 min at 5,600*g*, and the cells were suspended in 100 mL of 20 mM phosphate buffer (pH 6.0). Referring to the previous method [13], the conductivity of MRSA suspension with the intervention of azalomycin F_5a_ was determined in triplicate. Briefly, the MRSA suspension was divided into three equal ones, two of which contained, respectively, azalomycin F_5a_ to obtain concentrations of 0.5 MIC (2.0 *μ*g/mL) and 1.0 MIC (4.0 *μ*g/mL). The third one without azalomycin F_5a_ was used as blank control. Then, the conductivity of each suspension was, respectively, determined at 0, 20, 40, 60, 80, 120, and 180 min after azalomycin F_5a_ was added.

### 2.4. Adenylate Kinase Activity

Referring to the previous method [14], adenylate kinase activity was determined in triplicate using 96-well plates. In brief, fifty milliliters of MRSA cultures at exponential phase (approx. 1 × 10^8^ cfu/mL) was added to each well containing 50 *μ*L MHB with different concentrations of azalomycin F_5a_. The final concentrations of azalomycin F_5a_ in the wells of lines C to F were, respectively, 0.25, 0.5, 1.0, and 2.0 of MIC, and the wells of line B without azalomycin F_5a_ were used for blank controls. Next, the plates were incubated at 37°C for 6 h on a rotary shaker (220 rpm) and then were equilibrated for 30 min at room temperature. Finally, 100 *μ*L of adenylate kinase reagent (Lonza Rockland, Inc., USA) was added to each well according to the instruction of ToxiLight® BioAssay Kit and incubated 30 min at room temperature in darkness. The relative luminescence unit (RLU) of each well was measured using a SpectraMax M5 microplate reader.

### 2.5. Influences of MRSA Cell-Membrane Lipids on Azalomycin F_5a_ against MRSA

MRSA cells at exponential phase were collected by centrifugation at 4000 rpm for 20 min and washed three times in 10 mM Tris-HCl, pH 3.0. Then, MRSA cell-membrane lipids were extracted by modified Bligh-Dyer method [15]. Briefly, 15 mL of CHCl_3_-CH_3_OH (2 : 1, v/v) was added to 4 mL of MRSA suspension and then extracted on a vortex mixer for 30 min. Next, 5 mL of CHCl_3_ and 5 mL of 10 mM Tris-HCl (pH 3.0) were successively added to the mixture for further extraction of phospholipids. Finally, the organic phase was collected and concentrated in vacuum on a rotary evaporator to obtain MRSA cell-membrane lipids.

Next, MRSA cultures at exponential phase were diluted with MHB (1 : 100) for anti-MRSA activity test. With the intervention of 128 or 512 *μ*g/mL of MRSA cell-membrane lipids, the MICs of azalomycin F_5a_ against MRSA ATCC 33592 were, respectively, measured in triplicate on a 96-well plate using broth microdilution method [12, 16]. MHB that only contained azalomycin F_5a_ and 128 or 512 *μ*g/mL of MRSA cell-membrane lipids were, respectively, used as controls; the influences of MRSA cell-membrane lipids on azalomycin F_5a_ against MRSA were investigated.

### 2.6. Influences of DPPG on Azalomycin F_5a_ against MRSA

MRSA cultures at exponential phase (approx. 1 × 10^8^ cfu/mL) were diluted with MHB (1 : 100) for anti-MRSA activity test. With the intervention of 32 and 64 *μ*g/mL of 1,2-dipalmitoyl-*sn*-glycero-3-phosphoglycerol (DPPG) purchased from Avanti Polar Lipids, (USA), the MICs of azalomycin F_5a_ against MRSA ATCC 33592 were measured in triplicate on a 96-well plate using broth microdilution method [12, 16]. MHB that only contained azalomycin F_5a_ and 32 or 64 *μ*g/mL of DPPG were, respectively, used as controls; the influences of DPPG on azalomycin F_5a_ against MRSA were further investigated.

### 2.7. Statistical Analysis

All statistical analyses and diagrams were performed with Microsoft Office Excel 2007. The results were expressed as $\bar{x}\;\pm \;s$, and *t*-test was used for the comparison between two groups. *P* ≤ 0.05 indicates a significant difference between blank control and azalomycin F_5a_ group with specific concentration.

## 3. Results

### 3.1. Conductivity of MRSA Suspension

**Figure 2:**
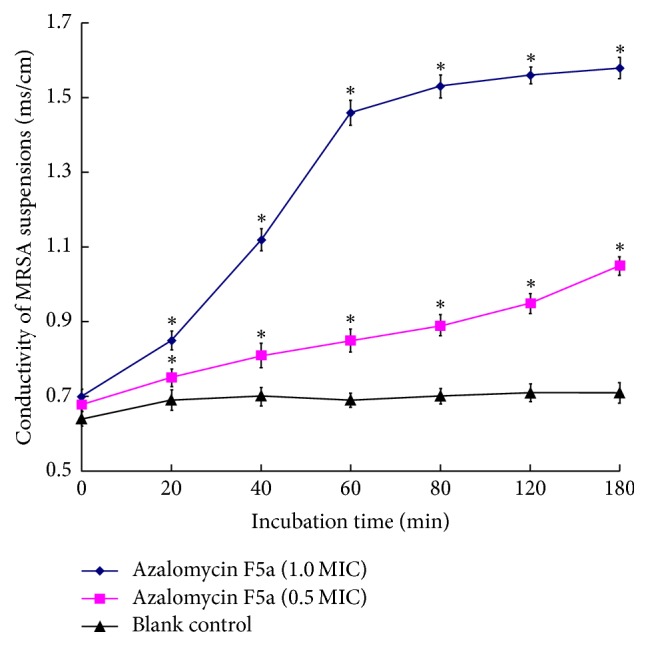
*Conductivities of MRSA suspensions treated with different concentrations of azalomycin F*_5*a*_. MRSA ATCC 33592 suspensions were, respectively, treated with 0.5 and 1.0 MIC of azalomycin F_5a_, and their conductivities (*n* = 3) were recorded at 0, 20, 40, 60, 80, 120, 160, and 180 min using a conductivity meter. *∗* indicates a significant difference between blank control and azalomycin F_5a_ group with specific concentration (*P* ≤ 0.05).

Treated with different concentrations of azalomycin F_5a_, the conductivities of MRSA suspensions were showed in Figure 2. Being relative to blank control, the conductivities of suspensions containing azalomycin F_5a_ significantly increased, while the increasing rates and final conductivities were different along with different concentrations of azalomycin F_5a_. Moreover, treated with 4.0 *μ*g/mL of azalomycin F_5a_, the suspension conductivity increased rapidly in 20 to 60 min, and the following conductivity tended to be stable. These indicated that the cellular substances of MRSA leaked rapidly in 20 to 60 min and finally led to the cell lysis of MRSA.

### 3.2. Adenylate Kinase Activity

**Figure 3:**
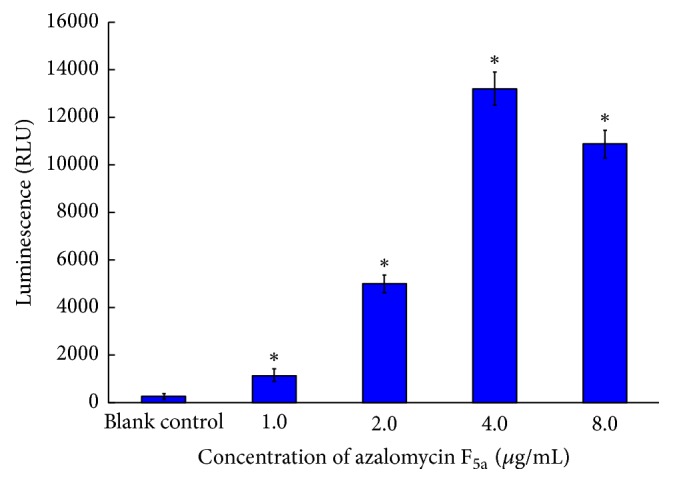
*Adenylate kinase activities of MRSA cultures treated with different concentrations of azalomycin *F_5a_. MRSA ATCC 33592 cultures, respectively, treated with 1.0, 2.0, 4.0, and 8.0 *μ*g/mL of azalomycin F_5a_, were incubated at 37°C for 6 h, and their luminescence (*n* = 3) was recorded using a SpectraMax M5 microplate reader. *∗* indicates a significant difference between blank control and azalomycin F_5a_ group with specific concentration (*P* ≤ 0.05).

The adenylate kinase activities of MRSA cultures treated with different concentrations of azalomycin F_5a_ were showed in Figure 3. The results indicated that luminescence remarkably increased as the concentration of azalomycin F_5a_ increased. The cultures presented highest luminescence when the concentration of azalomycin F_5a_ was 4.0 *μ*g/mL (equal to MIC), and this deduced that azalomycin F_5a_ could lead to the leakage of adenylate kinase from MRSA cell. Moreover, the luminescence decreased slightly when the concentration of azalomycin F_5a_ was 8.0 *μ*g/mL, which might be attributed to the decreased cell numbers at this concentration (2 MIC) of azalomycin F_5a_.

### 3.3. Influences of MRSA Cell-Membrane Lipids on Azalomycin F_5a_ against MRSA

The MICs of azalomycin F_5a_ against MRSA ATCC 33592 were, respectively, 16 and 32 *μ*g/mL with the intervention of 128 and 512 *μ*g/mL of cell-membrane lipids extracted from test MRSA, and that without the intervention of lipids was 4.0 *μ*g/mL. These indicated that the anti-MRSA activity of azalomycin F_5a_ could be weakened by cell-membrane lipids extracted from test MRSA and further deduced that there were some interactions between azalomycin F_5a_ and cell-membrane lipids.

### 3.4. Influences of DPPG on Azalomycin F_5a_ against MRSA

Both of the MICs of azalomycin F_5a_ against MRSA ATCC 33592 were 32 *μ*g/mL with the intervention of 32 and 64 *μ*g/mL of DPPG, while that without the intervention of DPPG was 4.0 *μ*g/mL. These indicated that the anti-MRSA activity of azalomycin F_5a_ could be weakened by DPPG and deduced that there were some interactions between azalomycin F_5a_ and DPPG.

## 4. Discussion

Considering that antibiotic resistance spread widely, the research and development of new antibiotics against multidrug-resistant organisms were desperate [9, 17]. After the planar structure and relative configuration of azalomycin F_5a_ were elucidated by us, its anti-MRSA and anti-VRE activities were discovered in our previous works [10, 12]. Inspired by the fact that azalomycin F could lead to the leakage of cellular substances of* B. subtilis* [4], the interactions between azalomycin F_5a_, as a representative of these compounds, and MRSA cell membrane were researched. The results showed that azalomycin F_5a_ could significantly increase the conductivity of MRSA suspensions when its concentration increased to 4.0 *μ*g/mL (equal to MIC). This indicated that a large amount of cellular substances leaked from MRSA cell treated with azalomycin F_5a_ and further deduced that that azalomycin F_5a_ killed MRSA likely by damaging cell membrane or increasing permeability. As adenylate kinase was an intracellular substance, the fact that extracellular adenylate kinase activity remarkably increased when the concentration of azalomycin F_5a_ increased also proved above inferences.

The MRSA cell membrane mainly contains lipids and proteins, and the former is an important factor for cell-membrane integrity, stability, and permeability. To explore the interactions between azalomycin F_5a_ and cell membrane, the anti-MRSA activity of azalomycin F_5a_ against MRSA ATCC 33592 was determined with the intervention of cell-membrane lipids extracted from test MRSA. The results showed the anti-MRSA activity of azalomycin F_5a_ could be weakened by cell-membrane lipids isolated from test MRSA strain. Thereby, some interactions between azalomycin F_5a_ and MRSA cell-membrane lipids were deduced, which possibly caused the molecular numbers of azalomycin F_5a_ interacting with the membrane lipids bilayer of MRSA to decrease. To confirmed this and discover more detailed information, the anti-MRSA activity of azalomycin F_5a_ against MRSA was further determined with the intervention of DPPG which is a main component of MRSA cell-membrane lipid [18]. Excitedly, the results indicated that the anti-MRSA activity of azalomycin F_5a_ could be significantly weakened by DPPG, which deduced that there were some interactions between azalomycin F_5a_ and DPPG. Thus, DPPG lying in cell membrane might be an important molecular target of azalomycin F_5a_ against MRSA.

The resistance mechanism of MRSA is very complex and mainly involves various proteins expressed from resistant genes [19, 20]. These proteins mostly embed in cell membrane, and their functions accordingly depend on the integrity and liquidity of cell membrane. As it is difficult for MRSA to modify the drug-resistant proteins lying in the cell membrane in a short time, new antibiotics targeting MRSA cell membrane become an important field on the research and development of anti-MRSA drugs [21]. Among them, daptomycin, a lipopeptide antibiotic approved by FDA in 2003, was generally used for treating infection caused by MRSA being resistant to vancomycin [22, 23]. It kills Gram-positive pathogens in a strictly calcium dependent manner by perturbing the integrity and electrochemical gradient of cell membrane [24]. Recent reports indicated that the change of cell-membrane lipid profiles was a key factor for the resistance of MRSA to daptomycin [18, 25]. Thereby, some interactions between azalomycin F_5a_ and cell-membrane lipids especially DPPG indicated that cell-membrane lipids especially DPPG might be an important molecular target of azalomycin F_5a_ against MRSA and that the molecular mechanism of azalomycin F_5a_ interacting with DPPG and cell-membrane lipids of MRSA was worth being further detailed using ^31^P NMR, ATR-FT-IR, DSC, and Raman technologies with the help of models of biological membranes and molecular dynamics simulation.
